# Polysaccharide-Based Aerogel Bead Production via Jet Cutting Method

**DOI:** 10.3390/ma11081287

**Published:** 2018-07-25

**Authors:** Imke Preibisch, Philipp Niemeyer, Yusuf Yusufoglu, Pavel Gurikov, Barbara Milow, Irina Smirnova

**Affiliations:** 1 Institute of Thermal Separation Processes, Hamburg University of Technology, 21073 Hamburg, Germany; pavel.gurikov@tuhh.de (P.G.); irina.smirnova@tuhh.de (I.S.); 2 Institute of Materials Research, German Aerospace Center, 51147 Cologne, Germany; philipp.niemeyer@dlr.de (P.N.); barbara.milow@dlr.de (B.M.); 3 Arcelik A.S., Central R&D Department, Istanbul 34950, Turkey; yusuf.yusufoglu@arcelik.com

**Keywords:** aerogel, biopolymer, pectin, alginate, chitosan, beads, jet cutting

## Abstract

The aim of this work is to develop a method to produce spherical biopolymer-based aerogel particles, which is capable for scale-up in the future. Therefore, the jet cutting method is suggested. Amidated pectin, sodium alginate, and chitosan are used as a precursor (a 1–3 wt. % solution) for particle production via jet cutting. Gelation is realized via two methods: the internal setting method (using calcium carbonate particles as cross-linkers and citric and acidic acid for pH adjustment) and the diffusion method (in calcium chloride solutions). Gel particles are subjected to solvent exchange to ethanol and consequent supercritical drying with CO_2_. Spherical aerogel particles with narrow particle size distributions in the range of 400 to 1500 µm and a specific surface area of around 500 m^2^/g are produced. Overall, it can be concluded that the jet cutting method is suitable for aerogel particle production, although the shape of the particles is not perfectly spherical in all cases. However, parameter adjustment might lead to even better shaped particles in further work. Moreover, the biopolymer-based aerogel particles synthesized in this study are tested as humidity absorbers in drying units for home appliances, particularly for dishwashers. It has been shown that for several cycles of absorption and desorption of humidity, aerogel particles are stable with an absorption capacity of around 20 wt. %.

## 1. Introduction

Biopolymer-based aerogels have become increasingly important for various applications in foods, pharmaceuticals, tissue engineering, catalysis, and cosmetics in the last decades [1,2,3,4,5]. In many of these applications, round-shaped particles and beads with narrow particle size distributions are advantageous for handling, processing, and application requirements. For instance, for pulmonary drug delivery, small particles (below 10 microns for porous aerogel particles) [6] are required for the drug transport to the lung. Therefore, spherical shape of the particles is desired for a good flowability. In food industry, hydrogel particles can be used to mimic the taste and mouth feel of fat emulsions with reduced fat content [7]. In many applications, millimeter-sized spherical aerogel beads are used in catalysis [8].

To produce aerogel particles or beads of different sizes, dripping and emulsion gelation methods are the most promising approaches. In the emulsion gelation method, the aqueous biopolymer solution is dispersed in an oil phase. Resulting emulsions are stabilized by using surfactants. Subsequently, gelation of the droplets (dispersed phase of the emulsion) is induced by addition of a solution of the gelling agent [9]. Principally, the scale-up of the emulsion gelation method for production of large amounts of aerogel particles is possible. One way could be a scale-up of the stirred vessel size for the production of biopolymer solution emulsion. Another possibility is the continuous production of emulsions (for instance, by a rotor–stator machine). After the gelation process, gel particles are separated from the continuous oil phase and are dried to be converted into aerogel particles.

Particle sizes of a few hundred microns up to several millimeters can be achieved with different variations of the dripping method, such as simple dropping and vibrated dropping [10]. Within the dripping method, biopolymer solutions are dropped into a gelation bath, where a gelling agent induces gelation [5]. Particle size depends mainly on the tube or nozzle diameter [10]. The dripping method delivers particles with narrow particle size distributions, but is quite work intensive and enables only limited throughput. Further, all dripping methods are limited by the viscosity of processed solutions. Thus, it is preferred to use multiplication of the number of nozzles than via scale-up of the whole process and apparatus for a scale-up of the dripping method [10].

So far, the production of biopolymer aerogel particles has been realized only as a batch process on a small scale and no scale-up approaches are demonstrated. However, to enable industrial applications, a new and scalable production technology for aerogel particles is required. Whereas, for the production of small aerogel particles (below 500 μm), the emulsion–gelation method described above seems to be promising [2,11] for larger particles, and an alternative enabling the high throughput (or continuous) production is needed. In this work, we suggested the jet cutting method for the production of biopolymer-based aerogel particles and beads as an alternative to dripping techniques.

Throughout the jet cutting method, a liquid jet of biopolymer solution is cut by a rotating cutting disc. Obtained liquid cylinders fall along their trajectory to the ground. Throughout falling, due to surface tension cylinders form spheres, liquid drops are collected at the end of the particle trajectory. So far, the jet cutting method is used for the production of inorganic and organic spherical beads for diverse pharmaceutical, agricultural, cosmetic and cleaning applications [10] (https://www.genialab.com/production/). Particle sizes can be set between a few hundred microns and up to several millimeters with different parameters, such as nozzle diameters, jet velocities, cylinder ratios and cutting frequencies [10]. Two scale-up methods for the jet cutting technology are possible: (1) multiplication of the number of nozzles, despite its drawbacks related to increased cutting loss, and (2) increase of cutting frequency to increase the number of produced particles. For the increase of cutting frequency several parameters, such as the throughput, it is necessary to adjust the jet velocity and cutting velocity simultaneously to maintain particle size and shape [10]. The jet cutting method overcomes the limitation of simple dripping methods regarding the viscosity limitation of processed solutions [10,12]. Adjustment of the jet cutting process can also enable processing of low viscous solutions [12]. Production of alginate beads via the jet cutting method has been reported before [12,13]; however, they were not converted into aerogels.

At the end of the droplet trajectory, gelation of biopolymer solutions can be achieved with different gelation methods, which are highly desirable for the jet cutting process in “bath-gelled” methods, such as acid induced gelation or ionotropic gelation [14,15]. When biopolymer solutions come into contact with the gelation bath admixed cross-linker or acids in the bath liquid, gelation progresses from the outside of biopolymer solution droplet to their center. Therefore, two general methods can be identified as: (1) the diffusion method and (2) the internal setting method [1]. In the diffusion method, the gelation-inducing agent (e.g., ions or acid) diffuses from the gelation bath into the biopolymer solution, inducing the gel formation from the outer layer of the droplet towards its core. Hence, the gelation rate is mainly controlled by the diffusion. For the internal setting method, the solid gelling agent is dispersed inside the biopolymer solution, gelation is triggered by the subsequent dissolution of the agent due to changes of the external conditions like pH value or temperature and occurs more homogeneously throughout the droplet [1]. 

In this work, both gelation methods were combined with the jet cutting in order to produce biopolymer-based aerogel particles from alginate, amidated pectin, and chitosan, respectively. The jet cutting method was adjusted to perform the gelation process directly in the collecting bath at the end of the droplet trajectory, and thereby the conditions allowing to avoid agglomeration of particles were identified. Strategies to perform the subsequent solvent exchange and the supercritical drying of the particles were also discussed.

After the production of biopolymer-based aerogel particles, it was aimed to test the aerogel materials as humidity absorbers in drying units for home appliances, particularly for dishwashers. Throughout the whole washing cycle of a regular dishwasher, the drying step consumed a considerable amount of total energy, which was about 45% of the overall energy consumption by heating up the water temperature to approximately 65 °C. It was possible to reduce the energy consumption by more than 20% by lowering the heating temperature or even eliminating the heating step from the drying cycle by using water vapor absorbers. The usability of biopolymer-based aerogels for the application of water vapor absorbers showed the potential of aerogels to reduce the energy consumption significantly, and could be the first usage of aerogels in this kind of application in the market. The outcomes of this study will help to extend the use of aerogels in other appliances, such as driers, refrigerators, or even in products of electronic industry, where humidity is an important concern towards the goal of corrosion prevention.

## 2. Materials and Methods 

### 2.1. Materials

Amidated pectin (29% of esterification (DE), 21% of amidation (DA)) was kindly provided by Herbstreith und Fox KG, Neuenbürg/Württ., Germany. Calcium carbonate (CaCO_3_, light, precipitated powder, *D*_50_ = 1 µm) was kindly provided by Magnesia GmbH, Lüneburg, Germany. Sodium hydroxide (NaOH), citric acid, and calcium chloride (CaCl_2_) were purchased from Th. Geyer GmbH & Co. KG, Lohmar, Germany. Denatured ethanol 99.8%, and pure ethanol 99.5% were obtained from Carl Roth GmbH and Co. KG, Karlsruhe, Germany, and carbon dioxide (CO_2_) with a purity of 99.9% was supplied by AGA Gas GmbH, Hamburg, Germany. Sodium alginate (Hydagen 558P) was provided by BASF SE, Ludwigshafen, Germany, and sodium alginate (BioChemica A3249) was purchased from AppliChem GmbH, Darmstadt, Germany. Glacial acetic acid was purchased from VWR Chemical, Langenfeld, Germany. Shrimp chitosan was purchased from Sigma Aldrich, Taufkirchen, Germany. All chemicals were used as received. Deionized water was used throughout the study.

### 2.2. Biopolymer Stock Solutions

Pectin stock solutions were prepared by dissolution of biopolymers in deionized water by using magnetic stirring at room temperature to reach different concentrations (1–3 wt. %). Part of the pectin solutions was further neutralized with 0.5 M sodium hydroxide to pH 7, and 0.18 g CaCO_3_/g pectin was added as a cross-linker for internal setting gelation. Solution homogenization was done with a rotor–stator machine (IKA Magic Lab, Staufen im Breisgau, Germany).

Alginate stock solutions of 1–3 wt. % were prepared by dissolution of alginate powder in deionized water. Stirring was performed with a dissolver stirring tool mounted on a high torque stirrer (Heidolph Hei-Torque 100, Schwabach, Germany). For experiments with internal setting method, 0.37 g CaCO_3_ per 1 g alginate were added and stirred until a homogeneous dispersion was achieved.

Chitosan stock solutions of 3 wt. % were prepared by dissolution of chitosan in 3 wt. % acetic acid with mechanical stirring.

### 2.3. Hydrogel Particle Production—Jet Cutting

**Figure 1:**
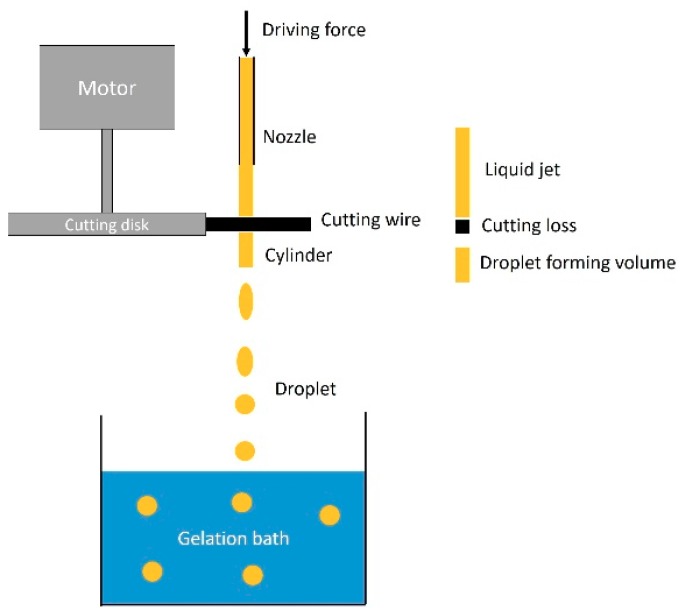
Schematic drawing of the jet cutting process.

Hydrogel particles were produced with the JetCutter Type S from geniaLab GmbH, Braunschweig, Germany. The extrusion of the biopolymer solution through the nozzle was driven by compressed air from the house supply line. A schematic drawing of the jet cutting process is shown in Figure 1. The throughput was tuned with a pressure-regulating valve. Nozzles with different diameters (250–1000 µm) were used to vary particle size. Experiments were carried out utilizing various combinations of nozzles, cutting discs, throughput, and cylinder ratios as well as cutting velocities. Two parameters (the nozzle diameter and cylinder ratio) were chosen to compare different cutting experiments. Used combinations of these parameters are shown in Table 1.

After cutting, liquid cylinders fell downwards into the collection bath. Throughout falling, cylinders formed spherical droplets due to the surface tension of the biopolymer solution. A suitable collection bath was placed at a distance of around 50 or 80 cm below the cutting tool. The composition of the gelation bath was adjusted to enable the gelation of the polysaccharide solutions. Used gelation methods and gelation baths for biopolymer solutions are shown in Table 2.

The volume of the gelation bath was at least five times the total volume of the processed biopolymer solution to enable good stirring and particle separation. After finishing the jet cutting, the content of the collection baths was stirred with a magnetic bar for at least ten more minutes to ensure complete gelation of particles and to avoid agglomeration. Gelled particles were removed from gelation bath via filtering. To avoid any loss of particles during collection, filter mesh sizes below nozzle diameters were chosen. Collected particles were transferred to the solvent exchange.

### 2.4. Solvent Exchange

Two ways of solvent exchange (water to ethanol) were performed on collected particles: (1) direct solvent exchange to 100 wt. % ethanol, (2) stepwise solvent exchange (30, 60, 90, 100 wt. % ethanol/20, 40, 60, 80, 100 wt. %) until a final ethanol concentration of 98 wt. % (respectively 98 vol.%) was reached inside the particles. Further, for some samples, an additional washing step with deionized water was performed before starting the solvent exchange to remove remaining components of the gelation bath from the particles, thus avoiding agglomeration during particle collection.

### 2.5. Supercritical Drying

After solvent exchange particles were taken out from the ethanol bath and packed into a filter paper, supercritical drying with CO_2_ was performed in an autoclave at a constant temperature of 60 °C and a pressure of 120 bar. Continuous flow of CO_2_ (20–80 g/min, respectively 240 g/min) was set until complete extraction of ethanol was done. Afterwards, slow depressurization of the autoclave (1–2 bar/min) was performed. Dried particles were collected from the autoclave and stored in sealed boxes until analysis.

### 2.6. Specific Surface Area

Specific surface areas of aerogel beads were measured via low temperature nitrogen adsorption/desorption (Quantachrome Nova 3000e, Odelzhausen, Germany and Micromeritics TriStar II 3020, Aachen, Germany) (BET).

### 2.7. Scanning Electron Microscope

Investigation of the inner structure of the resulting aerogels and particle size determination were done via a scanning electron microscope (SEM) analysis (Zeiss Supra VP55, Jena, Germany). Intact and cut particles were used for the study of outer and inner structure and particle size. Cutting of particles was done with a scalpel. Prior to analysis, all samples were sputtered with a thin layer (a few nanometers) of gold (Sputter Coater SCD 050, BAL-TEC) to avoid electrostatic charging during measurements. Such prepared samples were studied with the SEM under high vacuum at an accelerating voltage of 3 kV and magnifications between 1000 and 100,000 fold.

### 2.8. Optical Microscopy

Shape and size of obtained hydrogel and aerogel particles and beads were examined with an optical microscope (VisiScope TL384H) from VWR International GmbH, Darmstadt, Germany.

### 2.9. Humidity Absorption Test

**Figure 2:**
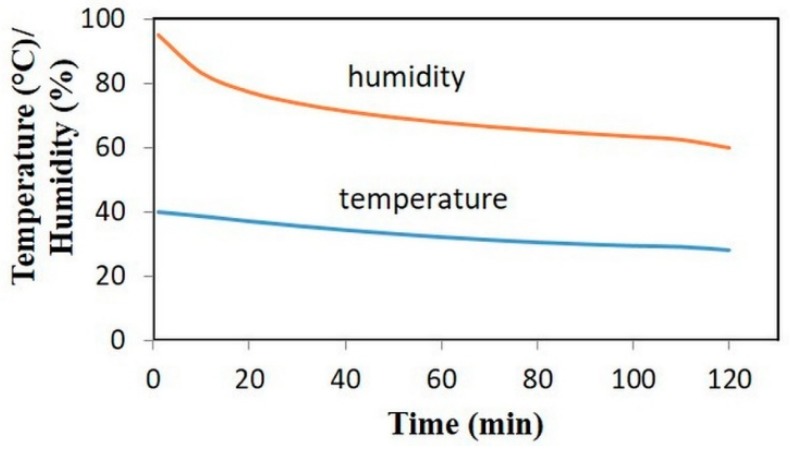
Program set in the humidity chamber simulating the relative humidity and temperature change in the dishwasher during the drying step.

The humidity absorption/desorption capacity of aerogel particles were tested in laboratory scale. For the determination of absorption capacities, aerogel samples were kept in a humidity chamber (Weiss Model, 2017) under two conditions: (a) 40 °C and 100% relative humidity (RH) and (b) 27 °C and 80% RH for three hours. Absorption capacity was determined in two ways: (a) samples were dried at 60 °C overnight and the weight of aerogel samples was determined after an absorption step and a drying step, respectively. The absorption capacity, $c_{abs},$ was calculated as a ratio between the difference of the final and initial aerogel weight and the initial aerogel weight (see Equation (1)); (b) evaluation of humidity uptake was done with a thermal gravimetric analysis (TGA, TA Instruments Q50, New Castle, DE, USA). Finally, samples were tested in real prototype dishwashers, and simulation with real conditions was performed. The changing conditions in the dishwasher prototype are shown in Figure 2. Here, the absorption capacity was determined by internal flow and energy consumption measurements of the prototype system.

The adsorption capacity in percentage was calculated as following:

(1) $$c_{abs}=\frac{w_{f}-w_{i}}{w_{i}}\times 100\%,$$

where $w_{i}\;\mathrm{and}\;w_{f}$ are the initial and final weight before and after adsorption tests, respectively. 

## 3. Results and Discussion

### 3.1. Jet Cutting Method

In this work, the jet cutting method was combined with two gelation mechanisms: the diffusion and internal setting methods (as described in the introduction). For both methods, the gelation in the collecting bath needs to be optimized, so that the droplets can form a gel without agglomeration or coalescence with other droplets/particles. Therefore, one of the most crucial parameters of the process is the gelation time.

High gelation rate might lead to an immediate gelation after the first contact of solution droplets with the gelation bath. Additionally, a low surface tension of the droplets might cause strong deformation during their hit on the gelation bath surface. Both factors might lead to a situation, when the droplet deformed by the hit on the surface of the bath`s liquid is “frozen” immediately by fast gelation. On the other hand, inappropriately long gelation time might cause deformation of liquid droplets or their coalescence during stirring in the gelation bath, thus leading to non-uniformity of particle size and shape. Depending on the polymer type, slow gelation rates might cause improper gelation after set gelation time, which results in the collection of only partly gelled particles, leading to a dissolution of non-gelled droplets during the washing step before solvent exchange. Therefore, the effects of the pH value of the solution, temperature, viscosity, and biopolymer concentration on the gelation rate should be accounted to understand their impact on the resulting particle shape. Another important parameter influencing the particle shape is the distance between the cutting tool and the collection bath, which needs to be properly adjusted.

In this work, all studied biopolymer solutions were processed with a table-top-sized JetCutter. The flow was induced with pressurized air. Pressures between 1 and 2 bar were sufficient to reach throughputs between 2 and 13 kg/h. Among the others, two important parameters of the jet cutting process are the cylinder ratio, which describes the ratio between the height and the diameter of the cut cylinders, and the nozzle diameter, which directly influences the jet diameter. The optimum values of these parameters for the production of spherical particles of certain size depend on the solution properties, such as viscosity and surface tension. Nevertheless, both parameters are not the only ones influencing the particle shape and size during the cutting process; the nozzle diameter is important to set the cylinder diameter, and therefore, strongly influences the particle size as well.

Thus, one of the goals of this work was to find the optimal cylinder ratio in combination with the nozzle diameter, which led to perfect sphericity of the particles and narrow particle size distribution.

Different cylinder ratios (cylinder height/cylinder diameter) were realized by tuning the nozzle diameter (from 250 to 1000 µm), the throughput, and the cutting frequency, as shown in Table 1. Therefore, jet velocities were calculated to be between 3.3 and 12 m/s, according to nozzle/throughput combinations used. Cutting of the jet caused a loss of biopolymer solution (a volume with height equal to the cutting wire diameter), as shown in Figure 1. This loss was accelerated in a vertical direction to the jet and collected in the so-called shield to avoid distribution. The cutting efficiency was determined from the amount of solution lost in the shield and depended on cutting frequency, jet velocity, and cutting angle. Experiments with small nozzle diameters below 500 µm often resulted in blocking of the nozzle. The same phenomenon was observed for the high biopolymer concentrations (3 wt. %), due to high viscosity and possible pre-gelation inside the jet cutting system. Stable jets of homogeneous flow and constant velocity and, therefore, constant volume flow could be obtained only from homogeneous solutions. Otherwise, inhomogeneity of viscosity or flowability resulted in moving jets of varying diameters, generating non-constant cylinder properties. 

### 3.2. Diffusion Method

The diffusion method of gelation could be applied to both aqueous solutions of pure pectin and pure alginate (2–3 and 1–3 wt. %, respectively).

Solutions of both biopolymers with different concentrations were jet cut and collected in a gelation bath containing an aqueous 0.5 wt. % CaCl_2_ solution. Free calcium ions diffuse into biopolymer solution droplets and interact with ionized carboxyl groups and hydroxyl groups of the polysaccharide chains, resulting in the formation of junction zones (“egg-box” model) and thus in gelation of the solution [16,17]. Even though the particles shape within one batch was not quite homogeneous, almost spherically shaped beads were observed for all combinations of the nozzle diameter and cylinder ratio. Obtained particles are shown in Table 3.

As can be observed from Table 3, in case of pectin, especially small cylinder ratios, resulted in well-shaped particles. Smaller cylinder ratios resulted in cut cylinders with dimensions, which were closer to the lateral dimensions of the targeted spherical droplet form. In this case, only small volumes were shaped by surface tension throughout falling, instead of large deformation for cylinders with a high cylinder ratio. In combination with small nozzle diameter, highest sphericity was obtained (see Table 3: a nozzle diameter and a cylinder ratio of pectin were 450 μm, and 0.7, respectively).

Larger nozzle diameter (1000 μm) results in mainly small, well-shaped particles comparable to those from smaller nozzles but also large, deformed particles indicating that breakdown of the large primary droplets into smaller secondary droplets occurred during the collection and stirring. The deformation and breakdown of large primary particles might be explained by the decreased stability of larger particles. Increasing nozzle diameter resulted in the increased particle volume. Due to the spherical shape of falling droplets with the increased droplet diameter, the volume (and mass) was increased with the diameter to the power of 3, whereas the cross-sectional area of the particles was increased by diameter to the power of 2. As a result, the ratio between the volume and cross-sectional area increased with the increased droplet diameter. This change has an impact on the droplet stability when hitting the surface of the collection bath and during stirring inside the bath. Increasing particle mass resulted in an increasing ratio of particle energy to particle surface, leading to less stability of the droplets. Therefore, larger particles were more likely broken down than smaller particles.

On the other hand, for both systems, larger particle diameters and deformed particles were obtained with larger nozzles, indicating deformation of unbroken droplets inside the gelation bath before proper gelation occurred. However, smaller nozzle diameter rarely resulted in deformed particles and narrower particle size distributions. This difference might be due to the larger impact of a gelled outer layer at small particles. For smaller particles, the ratio between the stable outer surface and the volume was smaller than that for larger particles. Therefore, a stable gelled layer at the outside was more effective for stabilization than for large particles, and might help to avoid deformation and destruction of particles during stirring. 

It has been found that high cylinder ratios generally led to deformed and elongated particles. Tails and flattening of the beads were observed, compared with 3 wt. % pectin, with a nozzle diameter of 500 μm and a cylinder ratio of 12.1 in Table 3. This might be explained by very slow transformation of cut cylinders into spherical droplets. A cylinder ratio of 12.1 meant cylinders were 12.1 times higher than their diameter, and therefore, particles had an elongated shape. When the droplets hit the surface of the gelation bath, it was likely that they were still elongated. In particular, for alginate particles, the combination of large nozzle diameters (500–600 nm) and high cylinder ratios (12.4 and 9.6) resulted in deformed particles. 

It is obvious that the nozzle diameter and cylinder ratio had a highly significant impact on the particle size and shape. Nevertheless, these two parameters are not sufficient to explain particle shape and size. It is likely that vigorous stirring needed for particle separation also causes damage of not yet gelled particles. More uniform flow fields and less turbulence inside the gelation bath could help to improve this situation. Therefore, further studies need to be done to evaluate the impact of these parameters on the particle size and shape.

**Figure 3:**
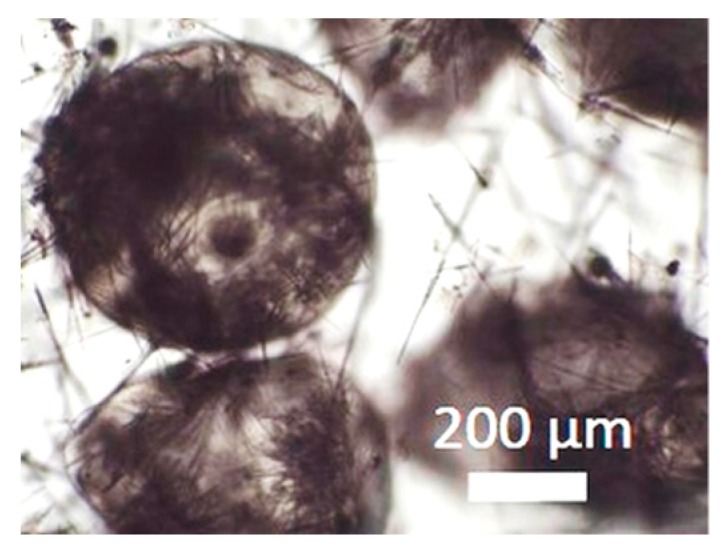
Collected pectin hydrogel particles from the diffusion method with solid needles.

During collection of pectin particles from a calcium chloride solution, solid needles were observed around the particles (Figure 3).

These needles were removed during washing, solvent exchange, and drying step. Most likely, these needles resulted from crystallized calcium chloride. Another observation for both polysaccharides is the formation of bubbles inside many of the particles, especially for spherical ones. The gas was slowly released during the solvent exchange before supercritical drying; nevertheless, generated bubbles resulted in particle inhomogeneity. One possible explanation for this phenomenon is that air was partly dissolved in the biopolymer solution during preparation. At the moment of extrusion through the nozzle, the pressure drop led to degassing of the gas from the solution, resulting in gas bubbles in the gelling particle. Another explanation might be that, during reshaping to spherical droplets from cut cylinders, air was included to the droplets. For the deformed particles, it was likely that the gas was released during the deformation and breakdown. The gas bubbles led to inhomogeneous structure of the particles and might influence their flowing and mechanical properties. 

### 3.3. Internal Setting Method

In case of the internal setting method, pectin and alginate solutions were mixed with solid CaCO_3_ particles, and gelation was induced during the contact with an acid in the gelation bath. For neutralized pectin solutions (2 and 3 wt. %, pH 7), an aqueous solution of citric acid (pH 3–4) was used as a collection bath, whereas 30 wt. % acetic acid was used for alginate. The drop of the pH value in the bath induced the dissolution of CaCO_3_ and ionotropic gelation of pectin and alginate took place as described in Chapter 3.2. In this case, the particle size of the dispersed solid CaCO_3_ was limit for the processing with the JetCutter. The used size of the nozzle was restricted by the particles with adequately small size, which were allowed to pass the nozzle. In our case, CaCO_3_ had an average size of ca. 1 μm. To avoid the blocking inside the nozzle due to agglomerates of CaCO_3_, appropriate homogenization of the dispersions with certain devices (e.g., a rotor–stator machine) was suggested.

Significant differences between pectin and alginate were observed at a low polymer concentration (1 wt. %). Pectin beads re-dissolved during the washing step due to incomplete gelation and weak particles. Further, after the collection in filter paper and supercritical drying particles with low pectin concentration (1 wt. %) were agglomerated. Pectin hydrogel particles from 2 and 3 wt. % solutions and alginate particles (from all used concentrations) were stable and overcame the washing step and solvent exchange without a visible damage. 

Gel particles obtained at different combinations of nozzle diameters and cylinder ratios for internal setting method are shown in Table 4. As can be seen, for the internal setting method, the impact of the cylinder ratio was less profound than for the diffusion method. In case of pectin solutions, a small cylinder ratio (0.9) resulted in spherical particles; however, for only slightly higher cylinder ratios (1–2), mainly badly shaped particles were obtained. For the larger nozzle diameter (500 μm), spherical particles could be reproduced for even larger cylinder ratios (4.7, 9.6). Spherical particles were found in nearly every gelation bath, indicating that the deformation of the particles might occur inside the gelation bath and not in the process of the droplet formation during falling. Especially for alginate solution, it was observed that high cylinder ratios (6.0–9.4) resulted in spherical particles for different nozzle diameters.

Generally, the internal setting gelation of pectin seemed to be slower compared to the diffusion method, which resulted in weaker gel particles after the same gelation time. In the internal setting method, contact with the gelation bath caused the drop of the pH value inside the droplets and subsequent dissolution of distributed CaCO_3_ particles. Calcium ions were released and induced gelation of pectin molecules in the whole droplet. Due to the required dissolution of CaCO_3_ particles, the gelation process seemed to be slower but led to more homogeneous particles, compared to the diffusion method. Nevertheless, it seemed that slower gelation favored deformation of droplets inside the gelation bath. Sheer forces due to stirring of the bath might cause the deformation of the droplets, whereas gelled particles might withstand these forces. 

In conclusion, both diffusion and internal setting methods are feasible to be combined with the jet cutting method to produce spherical-shaped aerogel particles from pectin and alginate solutions. However, the diffusion method is simpler in preparation and handling stock solutions in comparison to the internal setting method, where particle production is challenging due to the higher viscosities of the solutions and blocking of the nozzle with calcium carbonate. Further, especially for pectin, the diffusion method seems to be more favorable in terms of particle deformation and breakdown during the process. Therefore, a clear dependency of the particle shape and size on the cylinder ratio and nozzle diameter was observed. In case of the alginate solution, the internal setting method seems to be more independent of the nozzle diameter, regarding the particle shape. Nevertheless, spherical alginate particles could also be produced with the diffusion method. Taking into account this fact and also regarding the handling of the process, the diffusion method seems to produce spherical hydrogel particles more easily.

### 3.4. Aerogel Properties

**Figure 4:**
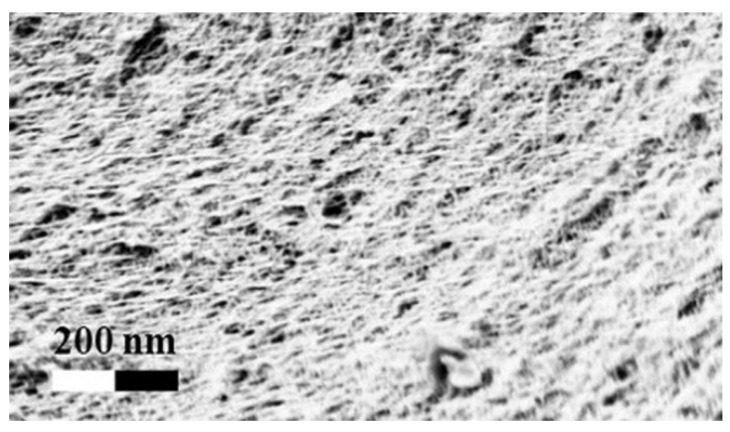
Inner structure of pure pectin aerogel produced via the diffusion method.

**Figure 5:**
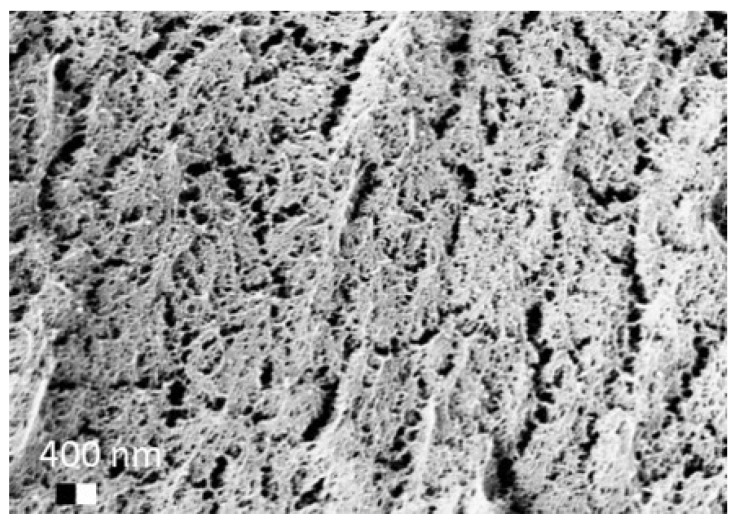
Inner structure of pure amidated pectin aerogel produced via the internal setting method.

**Figure 6:**
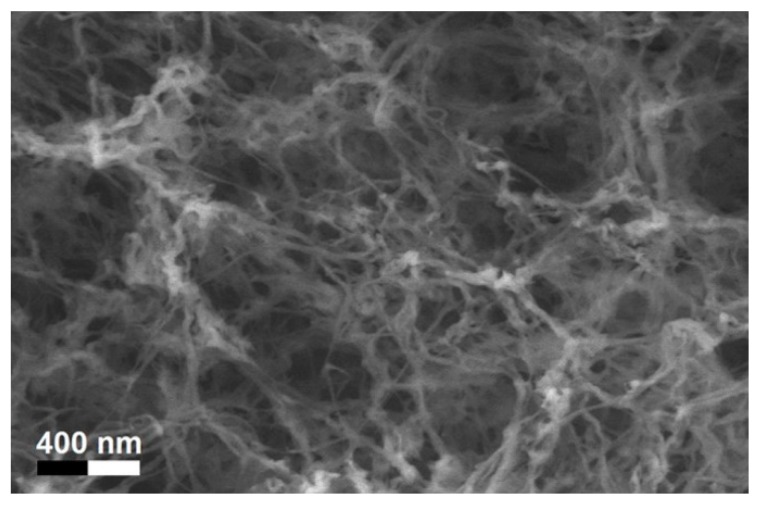
Inner structure of pure alginate aerogel produced via the diffusion method.

After solvent exchange and supercritical drying, aerogel particles were obtained. The specific surface areas of the obtained aerogel particles are shown in Table 5. Obtained specific surface areas were in the range of those of aerogel monoliths (1 wt. % pectin internal setting method with citric acid: 541±99 m^2^/g; 2 wt. % pectin internal setting method with carbon dioxide: 690±54; and 1 wt. % alginate internal setting method with carbon dioxide: 592±27 m^2^/g) produced in pre-studies. Aerogel particles presented, which were produced via emulsion gelation combined with diffusion and internal setting method in literature, showed alginate specific surface areas were around 394 m^2^/g by the diffusion method and 469–590 m^2^/g by the internal setting method [2,11] and 470–593 m^2^/g for pectin by the diffusion method [18]. Obtained specific surface areas of aerogel particles produced by the jet cutting method were in the same range as for monoliths and particles produced earlier. Further, the high specific surface area was in agreement with the SEM pictures (Figure 4, Figure 5 and Figure 6), showing highly porous microstructures of obtained aerogels by the jet cutting method. Therefore, we concluded that the jet cutting method is feasible to produce spherical aerogel particles from pectin and alginate solutions.

Similar specific aerogel surface areas were obtained with both internal setting and diffusion methods. 

### 3.5. Application of Aerogels as Humidity Absorbers in Drying Units of Dishwashers

**Figure 7:**
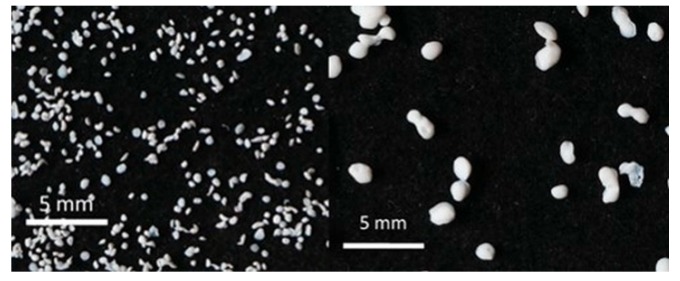
Chitosan aerogel particles (3 wt. %) via the jet cutting method: (**left**) a cylinder ratio of 5.3 and a nozzle diameter of 300 μm; (**right**) a cylinder ratio of 5.3 and nozzle diameter 700 μm.

**Figure 8:**
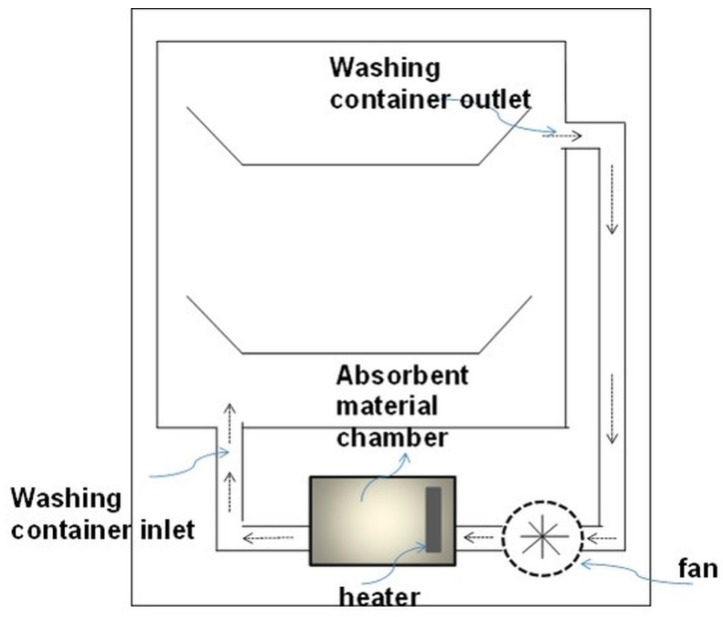
Schematic of a designed dishwasher for the prototype testing of aerogel particles.

Due to promising results for amidated pectin and alginate during the particle production with the jet cutting system and understanding of the parameters, chitosan aerogel particles were produced to demonstrate the high potential for the application in dishwashers (nozzle diameters: 300–700 μm, cylinder ratio: 5.3). Produced chitosan aerogel particles are shown in Figure 7. Synthesized chitosan aerogel particles were evaluated as humidity absorbers for the drying step in dishwashers. For this purpose, dishwasher prototypes were prepared. A schematic of a dishwasher designed for the prototype test of aerogel particles is shown in Figure 8. In the dishwasher prototype, which was developed in this study, the humid air is taken from the washing container to the absorption material chamber (with aerogel particles) by a fan. Aerogels inside the absorbent material chamber absorb the water, and dried air is transported to the washing container. Before the next washing cycle, regeneration of the aerogel particles takes place inside the absorbent material chamber. The temperature of humid air from the washing container is 45 °C and gradually decreases down to room temperature until the end of the cycle. RH at the beginning of the drying step is 100% and decreases to around 70% within five minutes and further down to 40% until the end of the cycle. A heating unit is attached to the absorbent material chamber for the regeneration of aerogel particles.

In order to select the best absorbent aerogel before incorporating them into the dishwasher, absorption/desorption capacities of the chitosan aerogel particles were characterized in laboratory conditions. Five different chitosan aerogel samples were tested. The aerogel samples were kept in the humidity chamber at 27 °C and 80% RH, which simulated the lowest temperature and RH conditions in a dishwasher during the drying step. The absorption/desorption cycles of the chitosan aerogel sample with the highest absorption capacity are shown in Table 6 (3 wt. % chitosan with a nozzle diameter of 700 μm and a cylinder ratio of 5.3). The average absorption capacity was found to be approximately 19 wt. %.

It was observed that absorption and desorption capacity was nearly constant over several cycles of absorption and desorption. First prototype tests resulted in consistent absorption capacities as those in laboratory scale.

This result is promising for the application of aerogel particles in dishwashers for the reduction of total energy consumption. Throughout the whole washing cycle in a conventional dishwasher, the drying step itself consumed a considerable amount of energy (45% of the overall energy consumption in a washing cycle) by heating up the water temperature to approximately 65 °C. This energy consumption could be reduced by lowering the heating temperature or even eliminating the heat step completely from the drying step by using water vapor absorbers instead. Tested chitosan aerogels showed the potential for the use as water vapor absorbers for the reduction of energy consumption.

## 4. Conclusions

Spherical biopolymer-based aerogels from amidated pectin, sodium alginate, and chitosan solutions were successfully produced via the jet cutting method and subsequent supercritical drying with CO_2_. It was shown that the jet cutting method could be combined with both the internal setting and the diffusion gelation method of polysaccharide solutions to obtain spherical hydrogel particles. One important parameter of the cutting process was identified to be the cylinder ratio, defined as the ratio between cylinder height and diameter. Cylinder ratios close to one resulted in spherical aerogel particles, whereas much higher cylinder ratios tended to result in deformed or broken particles. Structural analysis of the obtained aerogel particles revealed high specific surface areas, which were comparable to those of monolithic aerogels. Therefore, no negative effect of the jet cutting method on the aerogel properties was observed. For further optimization and understanding of the production process, the impact of the solution flow rate, cutting frequency, and the jet velocity during the cutting event on the particle size and shape will be studied in future work. 

Regarding the application of produced aerogel particles, it was shown that obtained chitosan aerogel particles showed a high humidity uptake capacity of around 20 wt. % when applied in an industrial prototype of a dishwasher. Spherical shape of particles was required due to easier handling and application in the prototypes. Therefore, aerogel particles produced via the jet cutting method are promising for the industrial application in this field. 

## Figures and Tables

**Table 1:** Nozzle/cylinder ratios used for jet cutting of different biopolymer solutions.

Solution	Nozzle Diameter (μm)/Cylinder Ratio				
Pure pectin in CaCl_2_, diffusion method	250/8.9	350/2.5–5.2	450/0.7–9.1	500/1.8–12.1	1000/0.6–2.1
Pectin/CaCO_3_-, acid induced internal setting	n.a.	n.a.	450/1.2–11.6	500/0.9–9.6	n.a.
Pure alginate in CaCl_2_, diffusion method	n.a.	300/10.1	400/9.8	500/12.4	600/9.6
Alginate/CaCO_3_-, acid induced internal setting	n.a.	300/8.7	n.a.	500/9.4	900/8.5

**Table 2:** Processed jet cutting experiments.

Biopolymer Solution	Gelation Method	Composition of Gelation Bath
Sodium alginate (1–3 wt. %)	Diffusion	Aqueous, 5 g/L CaCl_2_
Sodium alginate (3 wt. %)	Internal setting	Aqueous acetic acid (30 wt. %)
Amidated pectin (2–3 wt. %)	Diffusion	Aqueous 5 g/L CaCl_2_
Amidated pectin (1–3 wt. %), adjusted pH value to 7	Internal setting	Aqueous citric acid (pH 3–4)
Chitosan 3 wt. %	Diffusion	Aqueous 5 wt. % sodium hydroxide

**Table 3:** Used combinations of nozzle diameters and cylinder ratios for the production of gel particles via the diffusion method.

**Gel Particles Obtained from a 3 wt. % Amidated Pectin Solution by the Diffusion Method** (Nozzle Diameter/Cylinder ratio, Black Bar Corresponds to 200 μm, * 2 wt. % Amidated Pectin)			
250/8.9	350/2.5	350/2.6	350/4.3
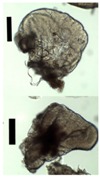	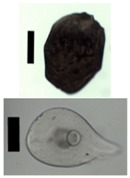	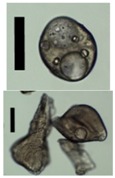	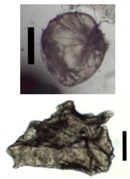
350/5.2	450/0.7	450/1.7	450/4.2
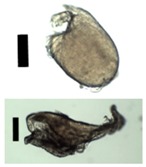	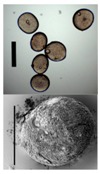	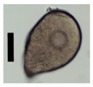	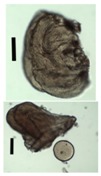
450/9.1	500/1.3	500/1.8	500/2.2
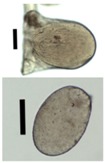	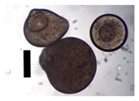	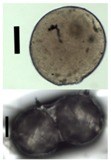	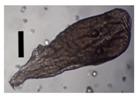
500/3.5	500/6.0	500/12.1	1000/0.6
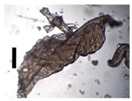	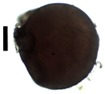	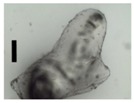	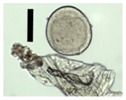
1000/2.1	* 450/1.5		
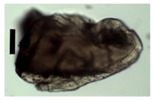	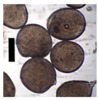		
**Gel particles obtained from a sodium alginate solution by the diffusion method** **(nozzle diameter/cylinder ratio, alginate concentration, white bar corresponds to 5 mm)**			
300/10.1, 1 wt. %	400/9.8, 2 wt. %	500/12.1, 1 wt. %	600/9.6, 2 wt. %
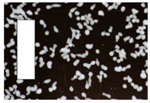	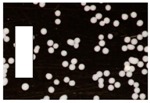	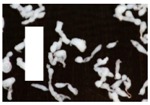	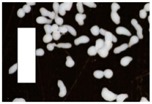

**Table 4:** Used nozzle diameters and cylinder ratios and obtained gel particles via the internal setting method.

**Gel Particles Obtained from a 2 wt. % Amidated Pectin Solution by the Internal Setting Method** **(Nozzle Diameter/Cylinder Ratio, Black Bar Corresponds to 200 μm)**			
450/1.2	450/1.6	450/1.9	450/3.4
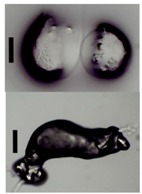	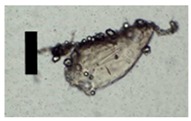	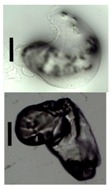	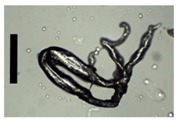
450/3.8	450/8.0	450/11.5	450/11.6
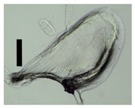	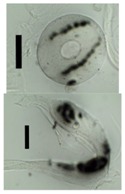	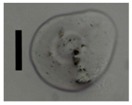	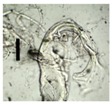
500/0.9	500/4.7	500/9.6	
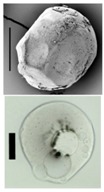	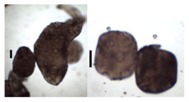	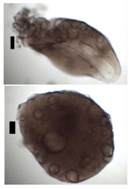	
**Gel particles obtained from a 3 wt. % sodium alginate solution by the internal setting method** **(nozzle diameter/cylinder ratio, alginate concentration, white bar corresponds to 5 mm)**			
300/8.7	500/6.0	500/9.4	
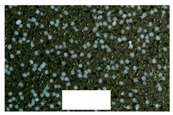	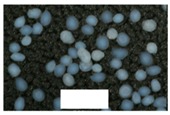	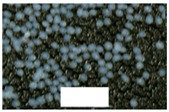	

**Table 5:** Average specific surface areas of produced aerogel particles.

Sample Preparation	Specific Surface Area (m^2^/g)	$\mathrm{Standard}\;\mathrm{Deviation}\;\left(n\geq 3\right)\;(m^{2}/g)$
3 wt. % Pectin in CaCl_2_ solution	528	58
2 wt. % pectin in CaCl_2_ solution	595	11
2 wt. % Pectin in citric acid	558	81
Alginate in CaCl_2_	537	2 (error of measurement apparatus)
Alginate in acetic acid	593	10

**Table 6:** Absorption and desorption behavior of 3 wt. % chitosan aerogel particles.

Sample	Cycle Number	Absorption (wt. %)	Desorption (wt. %)
3 wt. % chitosan aerogel particles	1	21.1	20.1
	2	19.3	17.9
	3	20.1	18.7
	4	19.8	18.1
	5	20.2	17.2
	6	19.3	17.1
	7	19.5	16.9
	8	18.9	17.1
	9	19.1	16.8
	10	19.6	16.1
	11	18.5	16.9
	12	18.9	16.8
	13	19.8	17.5
	14	20.1	17.8
	15	19.4	17.1
